# Preoperative oral antibiotic prophylaxis reduces *Pseudomonas aeruginosa* surgical site infections after elective colorectal surgery: a multicenter prospective cohort study

**DOI:** 10.1186/s12879-018-3413-1

**Published:** 2018-10-05

**Authors:** A Gomila, J Carratalà, J M Badia, D Camprubí, M Piriz, E Shaw, V Diaz-Brito, E Espejo, C Nicolás, M Brugués, R Perez, A Lérida, A Castro, S Biondo, D Fraccalvieri, E Limón, F Gudiol, M Pujol, on behalf of VINCat Colon Surgery Group, Xavier Serra-Aracil, Laura Mora, Antoni Cruz, Encarna Moreno, Francesc Aguilar, Lurdes Pagespetit, Núria Freixas, Albert Navarro, Lydia Martin, Camilo Sanz, Jordi Cuquet, Rosa Vazquez, Nares Arroyo, Ana Felisa Lopez, Simona Iftimie, Josefina Obradors, Anna Marrón

**Affiliations:** 1 Department of Infectious Diseases, Hospital Universitari de Bellvitge, Institut d’Investigació Biomèdica de Bellvitge (IDIBELL), Feixa Llarga s/n, 08907 L’Hospitalet de Llobregat, Barcelona, Spain; 2 VINCat Program, Barcelona, Spain; 3 University of Barcelona, Barcelona, Spain; 4 Department of General Surgery, Hospital General de Granollers, Barcelona, Spain; 5 Universitat Internacional de Catalunya, Barcelona, Spain; 6 Department of Infectious Diseases, Corporació Sanitària Parc Taulí, Barcelona, Spain; 7 Department of Infectious Diseases, Parc Sanitari Sant Joan de Déu, Barcelona, Spain; 8 Department of Infectious Diseases, Consorci Sanitari de Terrassa, Barcelona, Spain; 9 Department of Infectious Diseases, Hospital Universitari Mútua de Terrassa, Barcelona, Spain; 10 Department of Internal Medicine, Consorci Sanitari de l’Anoia, Barcelona, Spain; 11 Department of Internal Medicine, Fundació Althaia, Barcelona, Spain; 12 Department of Internal Medicine, Hospital de Viladecans, Barcelona, Spain; 13 Department of Internal Medicine, Hospital Universitari Sant Joan de Reus, Tarragona, Spain; 14 Department of General Surgery, Hospital Universitari de Bellvitge, Barcelona, Spain

**Keywords:** Healthcare-associated infection, Surgical site infection, Colorectal surgery, Colorectal cancer, Spain

## Abstract

**Background:**

Healthcare-associated infections caused by *Pseudomonas aeruginosa* are associated with poor outcomes. However, the role of *P. aeruginosa* in surgical site infections after colorectal surgery has not been evaluated. The aim of this study was to determine the predictive factors and outcomes of surgical site infections caused by *P. aeruginosa* after colorectal surgery, with special emphasis on the role of preoperative oral antibiotic prophylaxis.

**Methods:**

We conducted an observational, multicenter, prospective cohort study of all patients undergoing elective colorectal surgery at 10 Spanish hospitals (2011–2014). A logistic regression model was used to identify predictive factors for *P. aeruginosa* surgical site infections.

**Results:**

Out of 3701 patients, 669 (18.1%) developed surgical site infections, and 62 (9.3%) of these were due to *P. aeruginosa*. The following factors were found to differentiate between *P. aeruginosa* surgical site infections and those caused by other microorganisms: American Society of Anesthesiologists’ score III–IV (67.7% vs 45.5%, *p* = 0.001, odds ratio (OR) 2.5, 95% confidence interval (95% CI) 1.44–4.39), National Nosocomial Infections Surveillance risk index 1–2 (74.2% vs 44.2%, *p* < 0.001, OR 3.6, 95% CI 2.01–6.56), duration of surgery ≥75thpercentile (61.3% vs 41.4%, *p* = 0.003, OR 2.2, 95% CI 1.31–3.83) and oral antibiotic prophylaxis (17.7% vs 33.6%, *p* = 0.01, OR 0.4, 95% CI 0.21–0.83). Patients with *P. aeruginosa* surgical site infections were administered antibiotic treatment for a longer duration (median 17 days [interquartile range (IQR) 10–24] vs 13d [IQR 8–20], *p* = 0.015, OR 1.1, 95% CI 1.00–1.12), had a higher treatment failure rate (30.6% vs 20.8%, *p* = 0.07, OR 1.7, 95% CI 0.96–2.99), and longer hospitalization (median 22 days [IQR 15–42] vs 19d [IQR 12–28], *p* = 0.02, OR 1.1, 95% CI 1.00–1.17) than those with surgical site infections due to other microorganisms. Independent predictive factors associated with *P. aeruginosa* surgical site infections were the National Nosocomial Infections Surveillance risk index 1–2 (OR 2.3, 95% CI 1.03–5.40) and the use of oral antibiotic prophylaxis (OR 0.4, 95% CI 0.23–0.90).

**Conclusions:**

We observed that surgical site infections due to *P. aeruginosa* are associated with a higher National Nosocomial Infections Surveillance risk index, poor outcomes, and lack of preoperative oral antibiotic prophylaxis. These findings can aid in establishing specific preventive measures and appropriate empirical antibiotic treatment.

## Background

Currently, surgical site infections (SSIs) are the most frequent healthcare-associated infections (HAI) in acute-care hospitals in Europe and the US, accounting for 20% of all HAIs [1]. The development of an SSI lengthens patients’ hospital stay and increases readmission and mortality rates 2–11 times [2]. In particular, colorectal surgery is associated with high rates of SSI due to increased possibility of contamination during the procedure, although findings of SSI rates from studies considerably vary due to differences in the surveillance criteria used and the quality of data collection [3, 4].

*Pseudomonas aeruginosa* is one of the main causes of HAIs worldwide. Overall, it is considered to be the fourth leading cause of HAIs [1]; *P. aeruginosa* is frequently detected in patients with serious underlying conditions, and is associated with poor prognosis and high mortality [5]. Therapeutic options for *P. aeruginosa* infections are limited due to its intrinsic resistant pattern and its capacity to develop multiple drug resistance, necessitating the second-order or multiple antibiotic treatment [6, 7].

Despite the prevalence of SSIs among HAIs [1], the risk factors for *P. aeruginosa* in intraabdominal SSIs have not been examined in detail. Given the high frequency of elective colorectal surgery and the potential serious outcomes associated with *P. aeruginosa* infections, it is essential to determine the predictive factors of *P. aeruginosa* SSIs after colorectal surgery. The aim of this study involving a large, multicenter, prospective cohort of patients undergoing elective colorectal surgery was to identify specific predictive factors of *P. aeruginosa* SSIs, with special focus on the role of preoperative oral antibiotic prophylaxis, in order to propose specific preventive measures and appropriate empirical antibiotic treatment.

## Methods

### Setting and study patients

This was an observational, prospective cohort study of 3701 consecutive patients (age ≥ 18 years) who underwent elective colorectal surgery between January 2011 and December 2014 at 10 Spanish hospitals belonging to the VINCat Program [8]. VINCat is an HAI surveillance program based on the National Healthcare Safety Network (NHSN) model [9]. According to this program, hospitals submit information regarding patients’ demographics and comorbidities, procedure characteristics, microbiological and treatment data, as well as 30-day postoperative outcomes [10]. Post-discharge surveillance of SSIs until 30 days after surgery is mandatory and consists of a review of the electronic clinical records in primary and secondary care, checking of readmissions and emergency visits, and reviewing microbiological and radiological data [11]. For the purpose of this study, data prospectively collected from patients undergoing elective colorectal surgery and who developed SSI caused by *P. aeruginosa* and by other aetiologies were analysed. Patients with pre-existing infection at the surgical site at the time of surgery were excluded from the surveillance.

### Study variables

Variables included in this study are described elsewhere [12]. These variables included age, sex, American Society of Anesthesiologists’ (ASA) physical status, administration of mechanical bowel preparation (MBP), oral antibiotic prophylaxis (OAP), surgical risk index category based on the National Nosocomial Infections Surveillance (NNIS) modified system criteria [13], adequacy of the intravenous antibiotic prophylaxis, length of surgery (prolonged surgery was considered as the duration of surgery ≥75th percentile of the procedure), laparoscopic surgery, wound classification, date of SSI, site of infection (superficial and deep incisional SSI or organ-space (OS)-SSI, underlying disease (including neoplasia, inflammatory bowel disease and others), microbiology, and antibiotic treatment. Age, ASA score, and NNIS modified risk index were dichotomized for the analysis.

Study outcomes included duration of antibiotic treatment, length of stay (LOS), overall readmission, and overall mortality within 30 days of initial surgery. Readmission, if any, was included in the LOS.

### Definitions

SSIs were defined according to the Centers for Disease Control and Prevention (CDC) [14] into incisional (superficial and deep) and OS, and were stratified into categories of surgical procedures (− 1 to 3) according to the risk of surgical infection as defined by NHSN. Superficial and deep incisional SSI were considered together because the nature and management of these two types of infection is similar, in contrast to OS-SSI, which significantly differs. SSI due to *P. aeruginosa* was defined as the isolation of this microorganism from surgical samples.

The NNIS modified risk index predicts the risk of SSIs in colorectal surgery and range from − 1 to 2, depending on the presence of one or more of the following factors: ASA score III–V (1 point), contaminated or dirty-infected surgery (1 point), length of surgery ≥75^th^ percentile of the procedure (1 point), and laparoscopic surgery (− 1 point) [15]. This risk was calculated for all patients in our cohort.

The intravenous antibiotic prophylaxis included second-generation cephalosporin plus metronidazole administration, in accordance with the last consensus international guidelines on antimicrobial prophylaxis [16]. The treatment was deemed adequate, only when the antibiotics were administered according to the local protocol at each hospital, if the infusion was completed within 60 min of the surgical incision, and perioperative redosing administered (if indicated).

Administration of oral antibiotics in 2–3 doses a day before surgery was considered as OAP. In addition, patients received MBP and the intravenous antibiotic prophylaxis mentioned above. The use of OAP was not mandatory but based on the local protocol at each hospital. OAP included a combination of aminoglycoside (neomycin 1 g, gentamicin 80 mg, or kanamycin 1 g) with 1 g of metronidazole or 1 g of erythromycin [17].

The initial antibiotic treatment was either empirical or targeted, depending on the availability of microbiological sensitivity tests. The type and duration of antibiotic therapy was decided by the attending surgeon according to the local protocol. Source control was defined as any procedure which resolved the infection focus or repaired anatomical derangements. It was classified as reoperation when a new surgical procedure was performed, regardless of whether drainages were inserted or not. Drainage was considered when percutaneous or transrectal drainage was performed.

Treatment failure was defined as the persistence of clinical and/or radiological symptoms/signs of SSIs or all-cause mortality evaluated at 30 days post initial surgery.

### Microbiological studies

Surgical samples were collected in most patients (533/669) with suspected SSIs, and blood cultures were performed when indicated by the attending physician. Polymicrobial infection was defined as isolation of ≥2 microorganisms in surgical samples; however, with ≥3 microorganisms isolated, identification was not performed.

The microdilution method, according to the Clinical Laboratory Standard Institute (CLSI) guidelines, was used to test and interpret antibiotic susceptibility [18]. Multidrug-resistant phenotypes were screened according to the CLSI recommendations [19] and characterized by PCR and DNA sequencing. The multidrug-resistant gram-negative bacteria suspected were: (i) extended-spectrum beta-lactamase (ESBL)-producing Enterobacteriaceae; (ii) carbapenemase-producing Enterobacteriaceae; and (iii) multidrug-resistant strains of *P. aeruginosa*, resistant to at least three of the following classes of antibiotics: carbapenems, ureidopenicillins, cephalosporins (ceftazidime and cefepime), monobactams, aminoglycosides, or fluoroquinolones.

### Statistical analysis

Categorical variables were described as totals and frequencies while continuous variables were described as medians and interquartile ranges (IQR). Univariate analyses comparing patients with SSIs caused by *P. aeruginosa* and patients with SSIs caused by other microorganisms were performed using the chi-square test or Fisher’s exact test for categorical variables and the Mann–Whitney U test for continuous variables. A multivariate logistic regression analysis which included statistically significant and clinically relevant variables in the univariate analysis was performed to determine independent predictive factors of *P. aeruginosa* SSI. A *p* value of < 0.05 was considered to be statistically significant. Results were given as odds ratios (OR) and 95% confidence intervals (95% CI). The final model’s goodness-of-fit was assessed by the Hosmer–Lemeshow test. Data were analyzed using the IBM SPSS 20.0 (Chicago, Ill., USA).

## Results

**Fig. 1:**
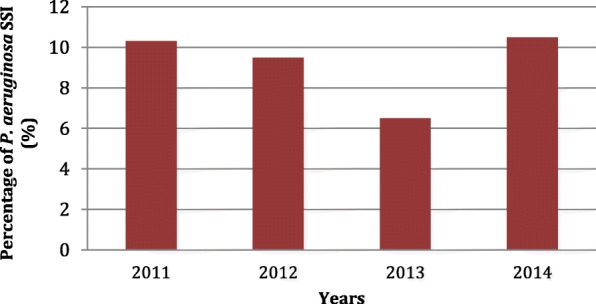
Number of SSI caused by *Pseudomonas aeruginosa* per year. *P. aeruginosa*: *Pseudomonas aeruginosa,* SSI: surgical site infections. (%): percentage. This figure shows the percentage of surgical site infections caused by *Pseudomonas aeruginosa* after elective colorectal surgery in the whole cohort of patients

Over the entire study period, 3701 patients were enrolled, and 669 (18%) developed SSIs. Of the 669 SSIs, there were 62 (9.3%) *P. aeruginosa* SSIs, 29 incisional SSIs, and 33 OS-SSIs. The number of *P. aeruginosa* SSIs remained stable over the 4-year study period, as shown in Fig. 1.

### Risk factor analysis

Patients with *P. aeruginosa* SSIs had higher ASA score III–IV (67.7% vs 45.5%, *p* = 0.001, OR 2.5, 95% CI 1.44–4.39), NNIS risk index 1–2 (74.2% vs 44.2%, *p* < 0.001, OR 3.6, 95% CI 2.01–6.56), longer duration of surgery (61.3% vs 41.4%, *p* = 0.003, OR 2.2, 95% CI 1.31–3.83), and less frequently received OAP (17.7% vs 33.6%, *p* = 0.01, OR 0.4, 95% CI 0.21–0.83) compared to patients with SSIs due to other organisms, as shown in Table 1.

**Table 1:** Risk factors analysis of patients with *P. aeruginosa* SSI and SSI due other organisms

Epidemiological characteristics	Non-SSI (*n* = 3032)	*P. aeruginosa* SSI (*n* = 62)	Other SSI (*n* = 607)	*P-value	OR (95% CI)
Age, median (IQR), years	69.6 (60.7–78)	71.3 (64.9–80)	69.9 (61.4–77)	0.1	1.0 (0.97–1.12)
Male sex, *n* (%)	1814 (59.8)	44 (71)	431 (71)	0.9	1.0 (0.56–1.78)
ASA III-IV, *n* (%)	1178 (38.9)	42 (67.7)	276 (45.5)	0.001	2.5 (1.44–4.39)
NNIS 1–2, *n* (%)	993 (32.8)	46 (74.2)	268 (44.2)	< 0.001	3.6 (2.01–6.56)
Indication for surgery, *n* (%):					
- Neoplasia	2868 (94.6)	57 (91.9)	577 (95.1)	0.3	0.5 (0.22–1.58)
- Inflammatory bowel disease	73 (2.4)	3 (4.8)	15 (2.5)	0.3	2.0 (0.56–7.13)
- Other	87 (2.9)	2 (3.2)	14 (2.3)	0.6	1.4 (0.31–6.36)
Type of surgery, *n* (%)				0.2	1.3 (0.81–2.33)
- Colon	2104 (69.4)	34 (54.8)	380 (62.6)		
- Rectum	928 (30.6)	28 (45.2)	227 (37.4)		
Adequate antibiotic prophylaxis, *n* (%)	2526 (83.3)	55 (88.7)	502 (82.7)	0.2	1.6 (0.73–3.37)
Duration of surgery ≥75th p^a^, *n* (%)	1163 (38.4)	38 (61.3)	251 (41.4)	0.003	2.2 (1.31–3.83)
Laparoscopic surgery, *n* (%)	1975 (65.1)	25 (40.3)	297 (48.9)	0.2	0.7 (0.41–1.29)
Detection of SSI during hospitalization, *n* (%)	_	46 (74.2)	435 (71.7)	0.6	1.1 (0.65–1.93)
Oral antibiotic prophylaxis, *n* (%)	1352 (44.6)	11 (17.7)	204 (33.6)	0.01	0.4 (0.21–0.83)
Mechanical bowel preparation, *n* (%)	2283 (77.1)	50 (80.6)	454 (75.8)	0.4	1.3 (0.69–2.56)
Ostomy, *n* (%)	715 (23.6)	29 (46.8)	218 (36)	0.09	1.5 (0.92–2.64)
Previous chemotherapy, *n* (%)	471 (15.5)	15 (24.2)	125 (20.6)	0.5	1.2 (0.66–2.26)
Previous radiotherapy, *n* (%)	452 (14.9)	14 (22.6)	112 (18.5)	0.4	1.3 (0.68–2.41)
Type of SSI, *n* (%):				0.6	1.1 (0.67–1.92)
- Incisional	_	29 (46.8)	304 (50.1)		
- Organ-space	_	33 (53.2)	303 (49.9)		

*P. aeruginosa: Pseudomonas aeruginosa*, *SSI:* surgical site infection, *IQR:* interquartile range, *ASA:* American Society of Anaesthesiologists’ physical status, *NNIS:* National Nosocomial Infections Surveillance Risk Index

*P-value refers to comparison between *P. aeruginosa* SSI and other SSI

^a^Length of surgery greater than the 75th percentile of the procedure

### Microbiological features

The comparison between patients with SSIs caused by *P. aeruginosa* and those with SSIs caused by other microorganisms is shown in Table 2. Of the 62 *P. aeruginosa* SSI cases, two had concomitant bacteremia (one case of *P. aeruginosa* and *Bacteroides fragilis,* and one of *Bacteroides spp)*. The SSIs caused by *P. aeruginosa* were more frequently polymicrobial (67.7% vs 33.4%, *p* < 0.001, OR 4.2, 95% CI 2.39–7.30) and less frequently accompanied by gram-positive organisms (16.1% vs 29.2%, *p* = 0.02, OR 0.4, 95% CI 0.23–0.94) than SSIs caused by other microorganisms. Multidrug-resistant *P. aeruginosa* was detected in three cases (4.8%). There were no differences in the number of multidrug-resistant *Enterobacteriaceae* isolated between patients with *P. aeruginosa* SSIs and those with SSIs due to other organisms.

**Table 2:** Microbiological features of SSI with or without *Pseudomonas aeruginosa*

Microorganisms	*P. aeruginosa* SSI (*n* = 62)	Other SSI (*n* = 607)	*P*-value	OR (95% CI)
Polymicrobial infection, *n* (%)	42 (67.7)	203 (33.4)	< 0.001	4.2 (2.39–7.30)
Gram-negative bacteria, *n* (%)	28 (45.2)	262 (43.2)	0.7	1.1 (0.64–1.83)
- *E. coli*	17 (27.4)	212 (34.9)	0.2	0.7 (0.39–1.26)
*• E. coli* MDR^a^	4 (6.5)	24 (4)	0.3	1.6 (0.56–4.99)
- *K. pneumoniae*	3 (4.8)	27 (4.4)	0.8	1.1 (0.32–3.70)
*• K. pneumoniae* MDR^a^	1 (1.6)	8 (1.3)	0.8	1.2 (0.51–9.97)
Gram-positive bacteria, *n* (%)	10 (16.1)	177 (29.2)	0.02	0.4 (0.23–0.94)
- Enterococcus spp	6 (9.7)	111 (18.3)	0.08	0.4 (0.20–1.13)
*• E. faecalis*	4 (6.5)	54 (8.9)	0.5	0.7 (0.24–2.02)
*• E. faecium*	2 (3.2)	56 (9.2)	0.1	0.3 (0.07–1.37)
- *S. aureus*	3 (4.8)	26 (4.3)	0.8	1.1 (0.33–3.86)
- *Coagulase negative staphylococci*	1 (1.6)	12 (2)	0.8	0.8 (0.1–6-35)
Fungus, *n* (%)	1 (1.6)	19 (3.1)	0.5	0.5 (0.06–3.85)
- *C. albicans*	1 (1.6)	15 (2.5)	0.6	0.6 (0.08–4.98)
Anaerobes, *n* (%)	1 (1.6)	33 (5.4)	0.2	0.3 (0.03–2.12)
- *B. fragilis*	0 (0)	16 (2.6)	0.1	0.9 (0.88–0.92)
- *C. perfringens*	0 (0)	3 (0.5)	0.6	0.9 (0.88–0.92)

*P. aeruginosa: Pseudomonas aeruginosa*, *SSI*: surgical site infection, *MDR*: multidrug-resistant, *E. Coli: Escherichia coli, K. pneumoniae: Klebsiella pneumoniae, A. baumannii: Acinetobacter baumannii, E. faecalis: Enterococcus faecalis, E. faecium: Enterococcus faecium, S. aureus: Staphylococcus aureus, C. albicans: Candida albicans, B. fragilis: Bacteroides fragilis, C. perfringens: Clostridium perfringens*

^a^*E. coli* MDR *and K. pneumoniae* MDR are included in the box above referring to the organism group

### Treatment

Among patients, 19 (65.5%) of 29 patients with *P. aeruginosa* incisional SSIs received antibiotic treatment, while all 33 patients (100%) with *P. aeruginosa* OS-SSIs received antibiotics. The initial antibiotic management of *P. aeruginosa* SSIs is shown in Table 3. Empirical treatment had a median duration of 10 (IQR 6–16) days and was switched to a targeted treatment in 33.3% of cases. In 13 cases (28.8%), there was no further treatment after empirical antibiotic. Targeted treatment, either initial or after the empirical regimen, had a median duration of 11 (IQR 7–18) days. Of the 33 patients with OS-SSI, 28 (84.8%) underwent source control of the infectious focus, 19 underwent reoperation due to significant anastomotic leakages while 9 underwent percutaneous drainage due to small leakages or abscesses.

**Table 3:** Initial antimicrobial management of *P. aeruginosa* SSI

Empirical	(*n* = 45, 72.5%)	Targeted	(*n* = 7, 11.3%)
Antibiotic	*n* (%)	Antibiotic	*n* (%)
Amoxicillin-clavulanic acid	15 (33.3)	Piperacillin-tazobactam	2 (28.5)
Meropenem/Imipenem	13 (28.8)	Meropenem	1 (14.2)
Piperacillin-tazobactam	9 (20)	3GC plus metronidazole	1 (14.2)
3GC	2 (4.4)	FQ	1 (14.2)
FQ plus metronidazole	2 (4.4)	3GC	1 (14.2)
Aminoglycoside plus metronidazole	1 (2.2)	FQ plus metronidazole	1 (14.2)
3GC plus metronidazole	1 (2.2)		
Piperacillin-tazobactam plus cotrimoxazole	1 (2.2)		
Antifungal			
Fluconazole	1 (2.2)		

*SSI*: Surgical site infection, *3GC*: Third-generation cephalosporin, *FQ*: fluoroquinolone

### Outcomes

Patients with *P. aeruginosa* SSIs underwent a longer duration of antibiotic treatment (median 17 [IQR 10–24] vs 13 [IQR 8–20] days, *p* = 0.015, OR 1.1, 95% CI 1.00–1.12), higher LOS (22 [IQR 15–42] vs 19 [IQR 12-28] days, *p* = 0.02, OR 1.1, 95% CI 1.00–1.17), and higher treatment failure rate (30.6% vs 20.8%, *p* = 0.07, OR 1.7, 95% CI 0.96–2.99) than patients with SSIs due to other organisms, as shown in Table 4. There was no difference in the mortality rate between the two groups.

**Table 4:** Outcome of patients with and without *P. aeruginosa* SSI

Outcomes	Non-SSI (*n* = 3032)	*P. aeruginosa* SSI (*n* = 62)	Other SSI (*n* = 607)	*P-value	OR (95% CI)
Duration of treatment, median (IQR), days	_	17 (10–24)	13 (8–20)	0.015	1.1 (1.00–1.12)
Treatment failure, *n* (%)	_	19 (30.6)	126 (20.8)	0.07	1.7 (0.96–2.99)
Readmission, *n* (%)	88 (2.9)	10 (16.1)	117 (19.3)	0.5	0.8 (0.39–1.63)
Length of readmission, median (IQR), days	(*n* = 88) 5 (3–9)	(*n* = 10) 11 (7–15)	(*n* = 117) 10 (7–15)	0.8	1.0 (0.91–1.06)
Length of stay, median (IQR), days	7 (5–10)	22 (15–42)	19 (12–28)	0.02	1.1 (1.00–1.17)
Mortality, *n* (%)	13 (0.4)	4 (6.5)	31 (5.1)	0.6	1.28 (0.43–3.75)

*P. aeruginosa*: *Pseudomonas aeruginosa*, *SSI*: surgical site infection, *IQR*: interquartile range

*P-value refers to comparison between *P. aeruginosa* SSI and other SSI

### Predictive factors

Multivariate logistic regression analysis of predictive factors for *P. aeruginosa* SSIs based on significant factors at the univariate analysis level is shown in Table 5. ASA score and duration of surgery, that were significantly associated with *P. aeruginosa* SSI in the univariate analysis, were not included in the multivariate analysis due to their association with NNIS risk index. The independent predictive factors for *P. aeruginosa* SSIs were NNIS risk index (OR 2.3, 95% CI 1.03–5.40) and preoperative OAP (OR 0.4, 95% CI 0.23–0.90).

**Table 5:** Multivariate analysis of predictive factors of *P. aeruginosa* SSI

	*P. aeruginosa* SSI/ Other SSI	*P*-value	OR (95% CI)
NNIS 1–2, %	74.2/44.2	0.04	2.3 (1.03–5.40)
Rectal surgery, %	45.2/37.4	0.3	1.4 (0.70–2.70)
Oral antibiotic prophylaxis, %	17.7/33.6	0.02	0.4 (0.23–0.90)
Ostomy, %	46.8/36	0.5	1.2 (0.60–2.30)

*P. aeruginosa*: *Pseudomonas aeruginosa, SSI*: surgical site infection, *OR*: Odds Ratio, *95% CI*: 95% confidence interval, *ASA*: American Society of Anaesthesiologists’ physical status. *NNIS*: National Nosocomial Infections Surveillance Risk Index

## Discussion

To the best of our knowledge, this is the first study to identify the clinical characteristics and risk factors of *P. aeruginosa* SSIs in a large cohort of patients undergoing elective colorectal surgery. The main findings are that NNIS modified risk index and OAP are associated with the risk of development of SSIs caused by *P. aeruginosa*.

Majority of the patients in our cohort had colorectal cancer. The intestinal microbiota of these patients present specific characteristics, showing an increased proportion of gram-negative bacteria, especially Enterobacteriaceae [20, 21]. However, *P. aeruginosa* does not seem to play a relevant role in the intestinal microbiota of patients, even with colorectal cancer. For this reason, we did not expect to detect a high rate of *P. aeruginosa* SSIs; however, we observed a rate of almost 10% in our cohort. A partial explanation could be that the systemic antimicrobial prophylaxis produced a selective antibiotic pressure leading to overgrowth of *P. aeruginosa*. Furthermore, tissue trauma and blood loss following a major surgery as well as the use of drugs (such as opioids) are associated with significant loss of diversity and abundance of the gut normal microbiota. This leads to an increase in the number and virulence of low-abundance collagenase-producing intestinal microorganisms, such as *Enterococcus faecalis* or *P. aeruginosa,* which may favor SSI and ileus by modulating the immune response of the host [22, 23]. OAP has been associated with good postoperative outcomes, nevertheless, the underlying changes in the gut microbiota are not completely known.

Previous studies have reported rates of *P. aeruginosa* SSI similar to those observed in the present study, despite the differences in patient characteristics (including emergency surgery, intensive care unit admission, and prior use of broad-spectrum antibiotics) [24, 25]. Patients with *P. aeruginosa* SSIs in our cohort had higher ASA score and NNIS risk index, longer duration of surgery, and lower levels of OAP. The study conducted by Montravers et al. [26], which involved more than 300 patients with community-acquired and nosocomial intraabdominal infections, revealed that *P. aeruginosa* was more frequently isolated in nosocomial cases (in more severely ill patients).

It should be noted that *P. aeruginosa* SSIs were more frequently polymicrobial in nature than SSIs caused by other organisms, as previously observed [24, 25]. It is possible that the interaction of *P. aeruginosa* with other gram-negative bacteria led to this clinical impact. We observed a very low rate of multidrug-resistant *P. aeruginosa*, explained by the short hospital stay of patients before surgery and the absence of prior long-term antibiotic therapy.

Among patients with *P. aeruginosa* SSIs, the most frequently used empiric antibiotic treatment failed to target the organism. This suggests that the attending physicians might not have considered *Pseudomonas* as the causative agent. The role of the empiric antibiotic treatment in the outcome of patients with intraabdominal infections has been widely discussed [24, 26]; however, as we noted previously [12], it is generally accepted that the control of the source of infection is the cornerstone of management in severe cases [27, 28]. Most patients with *P. aeruginosa* OS-SSIs in our cohort underwent source control.

Patients with *P. aeruginosa* SSIs had a longer antibiotic treatment, higher treatment failure, and longer hospitalization than patients with SSIs caused by other organisms. This reinforces the idea that *P. aeruginosa* affects patients with more serious underlying diseases and implies worse prognoses. However, we did not observe differences in mortality rates between patients with *P. aeruginosa* SSIs and SSIs caused by other organisms, probably due to our low overall mortality rate, neither did they observe differences, in studies previously cited [24, 26]. As reported previously, treatment failure among patients with the most serious SSIs in our cohort was not associated with any microbiological etiology, including *P. aeruginosa* [12].

The administration of OAP was a strong protective factor against the development of *P. aeruginosa* SSIs. Two previous outstanding studies [29, 30] based on the large American College of Surgeons National Surgical Quality Improvement Program (ACS-NSQIP) database, showed a significant decrease in the rates of postoperative incisional SSI, anastomotic leakage, ileus, and 30-day mortality in patients undergoing elective colorectal procedures who received MBP and OAP (compared to patients who had received MBP or OAP alone, or those who had not received any preparation). We also showed a reduction in the OS-SSI rate with the use of MBP combined with OAP [31]. Some authors have however suggested the same benefit in the use of OAP without MBP [32], but this need to be validated in further large multicenter randomized controlled trials.

The most appropriate combination of oral antibiotics has not been clearly stated. In our study, the most frequently used aminoglycoside was neomycin, since its poor absorption in the digestive tract allows all its effects to be concentrated in the intestinal lumen. This specific characteristic, which also rules it out for the treatment of systemic infections, may justify its good activity against *P. aeruginosa*. Although the use of OAP in elective colorectal surgery has been recommended in recent World Health Organization guidelines [33, 34], many hospitals have abandoned this practice over the last decade since MBP has been shown to be ineffective [35]. Since OAP is administered together with MBP, the use of OAP was also abandoned. Although evaluation of OAP was not an objective in our study, our results reinforce the use of OAP combined with MBP in reducing *P. aeruginosa* SSI rates.

This study has some limitations. First, the hospitals in our study differed in terms of size, characteristics, levels of activity, and type of preoperative oral preparation. As previously mentioned OAP was not administered in a uniform manner but according to local protocols that did not depend on the baseline characteristics of patients. However, all hospitals followed the VINCat recommendations and CLSI microbiological guidance. Second, because of the nature of our study, we could not exclude bias related to risk factors not included in the study. However, the large number of patients and the consistent collection of the data by expert infection control staffs, support the results.

## Conclusions

SSIs due to *P. aeruginosa* after elective colorectal surgery mainly occur in patients with a high NNIS risk index and in those who do not receive OAP. We recommend empirical antibiotic treatment covering the multi-susceptible *P. aeruginosa* in more severely ill patients who develop SSIs but do not receive OAP. We observed worse outcomes in patients with *P. aeruginosa* SSIs, as demonstrated by the need for longer antibiotic treatments, higher treatment failure, and higher LOS. Further studies are needed to prove the effectiveness of OAP in the prevention of *P. aeruginosa* SSIs after colorectal surgery.

## Abbreviations

95% CI: 95% confidence interval
ACS-NSQIP: American College of Surgeons National Surgical Quality Improvement Program
ASA: American Society of Anaesthesiologists
CDC: Centers for Disease Control and Prevention
CLSI: Clinical Laboratory Standard Institute
ESBL: Extended-spectrum beta-lactamase
HAI: Healthcare-associated infection
IBM SPSS: International Business Machines Corp. Statistical Package for the Social Sciences
IQR: Interquartile range
LOS: Length of stay
MBP: Mechanical bowel preparation
NHSN: National Healthcare Safety Network
NNIS: National Nosocomial Infection Surveillance
OAP: Oral antibiotic prophylaxis
OR: Odds ratio
OS-SSI: Organ-space surgical site infection
SSI: Surgical site infection
USA: United States of America
